# Refocusing Moving Ship Targets in SAR Images Based on Fast Minimum Entropy Phase Compensation

**DOI:** 10.3390/s19051154

**Published:** 2019-03-07

**Authors:** Xiangli Huang, Kefeng Ji, Xiangguang Leng, Ganggang Dong, Xiangwei Xing

**Affiliations:** 1 State Key Laboratory of Complex Electromagnetic Environment Effects on Electronics and Information System, National University of Defense Technology, Changsha 410073, China; huangxiangli_2008@126.com; 2 School of Electronic Science, National University of Defense Technology, Changsha 410073, China; luckight@163.com; 3 National Laboratory of Radar Signal Processing, Xidian University, Xi’an 710071, China; dongganggang@xidian.edu.cn; 4 Beijing Institute of Remote Sensing Information, Beijing 100192, China; xingxiangwei@nudt.edu.cn

**Keywords:** synthetic aperture radar (SAR), inverse synthetic aperture radar (ISAR), moving ship, refocusing, fast minimum entropy

## Abstract

Moving ship targets appear blurred and defocused in synthetic aperture radar (SAR) images due to the translation motion during the coherent processing. Motion compensation is required for refocusing moving ship targets in SAR scenes. A novel refocusing method for moving ship is developed in this paper. The method is exploiting inverse synthetic aperture radar (ISAR) technique to refocus the ship target in SAR image. Generally, most cases of refocusing are for raw echo data, not for SAR image. Taking into account the advantages of processing in SAR image, the processing data are SAR image rather than raw echo data in this paper. The ISAR processing is based on fast minimum entropy phase compensation method, an iterative approach to obtain the phase error. The proposed method has been tested using Spaceborne TerraSAR-X, Gaofeng-3 images and airborne SAR images of maritime targets.

## 1. Introduction

Synthetic aperture radar (SAR) is widely employed in military surveillance, geography mapping and resource surveying. High-resolution SAR image is of great significance for homeland and military security [1,2,3,4]. A moving ship is not a static target during image formation, and the SAR imaging results are blurred and defocused [5,6,7]. It is necessary to refocus the defocused ship for ship recognition, and precise motion compensation becomes a key element of refocusing.

The motion between the moving target and the radar contains translation and rotation motions [8,9]. Translation motion is the main cause of image defocusing. Motion compensation can eliminate the translation motion affection on image. It is a significant step of inverse synthetic aperture radar (ISAR) processing for refocusing moving target. Motion compensation commonly includes two steps. The first is range alignment which is coarse compensation [10,11]. The second is phase compensation which compensates the Doppler frequency shift caused by movement [1,5]. Range alignment and phase compensation are also referred to as autofocusing. Due to imperfection of coarse compensation, this paper focuses on phase compensation.

Methods for phase compensation may be divided into three categories. The first category is scatter-based algorithms, such as dominant scatter processing (DSP) method [12,13], phase gradient autofocus (PGA) method [14,15,16]. The DSP method is intuitive in concept and easy to implement, but it needs high-quality prominent point in echo, otherwise the image is inferior [13]. The PGA method estimates phase error with part of data selected by isolating defocused targets via center shifting and window operations. The disadvantage of PGA is that the imaging is sensitive to the selection of the dominant scatters, window length, and the iteration times [15]. The second category is optimization algorithms, including Doppler centroid tracking (DCT) method [17,18], maximum contrast (MC) method [19,20], maximum likelihood (ML) method [21,22] and minimum entropy (ME) method [23,24]. DCT is a classical autofocus method with good robustness and a small amount of computation. However, only the rotational motion is ignored, the DCT approach can provide the maximum likelihood estimation of the phase error. When one scatter or multiple scatters are located in a range bin, the motion compensation accuracy will decrease [18]. The MC method assumes a mathematical model for the received signal. The model parameters (target radial velocity, acceleration) are achieved by the maximum contrast criterion of the image. It should be pointed out that the method requires two-dimensional search parameters at the same time and the computation is large [19]. The ME method which is based on the overall information of image takes the entropy as the cost function. The focused image can be achieved by numerical iterative until minimum entropy are acquired. It has well performance under low signal noise ratio (SNR), while the efficiency is lower and the computation time is large [23]. The last category is other algorithms, such as spare representation (SR) method [25]. A sparse metric is defined to iteratively estimate the sparse scatterer coefficients and phase errors, while the SR method is proposed to only deal with autofocus issues and cannot simultaneously obtain high-resolution images. The scatter-based algorithms are based on the processing of dominant scatter center and pay attention to the phase history of isolated scatter center. The optimization algorithms obtain phase error via image quality evaluation. The image contrast and entropy are maximal and minimum respectively when the image are well-focused. The scatter-based algorithms are higher computational efficiency than optimization algorithms, but the image quality constructed with former algorithms are worse than latter algorithms results. The previous methods all have some drawbacks and are mainly applied in raw echo data.

There are two issues involved in processing raw echo data. One is that the aforementioned methods are often applied to raw echo data, while the moving ship’s position is hard to ascertain in raw data [6]. Hence those methods may have to cope with the entire raw dataset. The invalid data would occupy a massive amount of computation time when processing all the raw data. The other is that there may be a few ship targets with different motions in the raw data. Raw data is repeatedly processed for specific parameters of different moving ship, and the computation cost greatly increases [6]. The processing data are sub-images selected from ordinary SAR images, not raw data, and the sub-images are converted into the raw echo data domain by an inversion algorithm. The sub-images in the raw echo data domain are refocused with the ISAR technique, and the moving ships can then be well-focused. This thought has the advantage of easily locating moving ships in SAR images and the data size of sub-image is also smaller than the entire raw data.

To deal with these problems, a refocusing method for moving ships based on fast minimum entropy phase compensation is proposed in this paper. The processing data are sub-images containing moving ships rather than the raw echo data, and the ISAR technique is based on fast minimum entropy phase compensation. The refocusing method has three advantages, the first is that the computational burden is low, due to the smaller data size of the sub-image than the raw echo data. The second is the procedures of the inversion algorithm and image reconstruction are simple. The last is that the ISAR technique based on fast minimum entropy phase compensation has good image quality and computational efficiency.

The remainder of this paper is organized as follows: in Section 2, basic procedures of moving ship refocusing in SAR images is presented; ISAR processing based on fast minimum entropy phase compensation is elaborated in Section 3. In Section 4, experiments based on real SAR images are performed and the conclusions are presented in Section 5.

## 2. Basic Procedures of Moving Ship Refocusing in SAR Images

**Figure 1:**
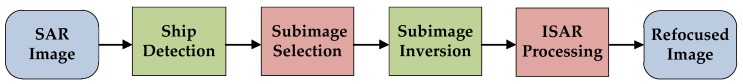
Block scheme of defocused target refocusing in SAR image.

Moving ships are regularly blurred and defocused in SAR images due to the translation motion with respect to the scene center. ISAR technology is a common method to form well-focused images, but it has two basic problems. One is that SAR images contain a very large number of ships, each with its own motion. Hence, it needs sub-images which only contain a single ship target. The other is the input of the ISAR processor is raw data, not SAR images, so it needs to get input data for the ISAR system which can serve as raw data. The basic procedures of refocusing moving ship in SAR image are represented in Figure 1.

The detailed procedures are as follows:

(a) Input a single look complex (SLC) image;
(b) Implement ship detection with software;
(c) Select sub-images, where each sub-image includes only a single defocused ship and has the same spatial resolution as the original image;
(d) Invert the sub-image to the equivalent raw data domain via an inversion method [26,27,28,29];
(e) Exploit ISAR processing to generate a focused image of the ship.

The sub-image needs to be inverted to equivalent raw data-like data containing only the target echo, background and residual clutter. The common inversion method is known as the range Doppler inversion [26].

**Figure 2:**
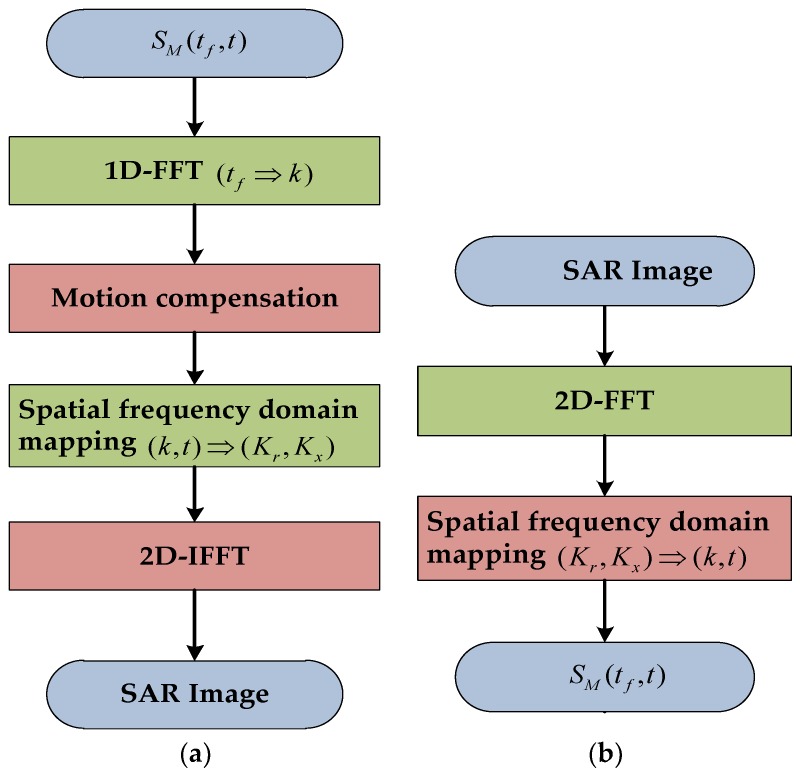
RD and IRD algorithm flow charts. (**a**) RD algorithm flowchart; (**b**) IRD algorithm flowchart.

When the angle variation is not too large, and the rotation vector is sufficiently stable during radar imaging, the range Doppler (RD) algorithm is applied for reforming SAR or ISAR images with high accuracy after motion compensation. The polar grid in the spatial frequency domain can be taken as a nearly regularly sampled rectangular grid with no need for interpolations. The main advantage of this method is the low amount of computation and it is the reason why it has been employed in many references [26,29]. The disadvantage is that the RD algorithm can only be used on low resolution SAR images when the spatial resolution is of the order of meters. The RD reformation algorithm is used when the SAR image is StripMap data. In this paper, the inverse range Doppler (IRD) algorithm is applied via a two-dimensional fast Fourier transform (FFT). The flowcharts of the RD algorithm and inverse RD algorithm are shown in Figure 2.

## 3. ISAR Processing Based on Fast Minimum Entropy Phase Compensation

We propose an ISAR processing method based on fast minimum entropy phase compensation to refocus sub-images of moving ship targets. The approach is a RD algorithm exploiting a Fourier transform to create the image. The fast minimum entropy phase compensation method is a non-parametric method and can be applied to arbitrary targets, even high-order polynomial models.

### 3.1. Signal Model

**Figure 3:**
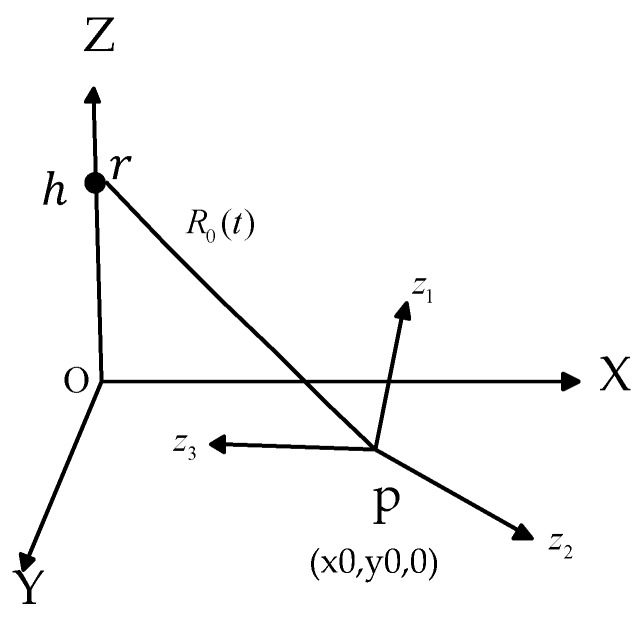
Geometry of the ISAR system.

The geometry of the ISAR system is depicted in Figure 3, where the radar is located at r(0, 0,h) in the (X, Y, Z) coordinates system. The reference coordinates system ($z_{1}$,$z_{2}$,$z_{3}$) is set at the target point p(x0, y0, 0). The distance between the radar and the target is $R_{0}(t)$. The back-scattering property of the target is represented by ξ(z) and z is the vector that locates a generic scatter point on the reference coordinate system. The received signal from the moving target can then be written as follows [30,31]:

(1) $$S_{R}(f,t)=rect(\frac{t}{T_{obs}})rect(\frac{f-f_{0}}{B})e^{-j\frac{4\pi f}{c}R_{0}(t)}\int_{V}\xi (z)e^{-j\frac{4\pi f}{c}(z^{T}•i{}_{R_{0}(t)})}dz$$

where $f_{0}$ is the carrier frequency, B the signal bandwidth and $T_{obs}$ the observation time. $i_{R_{0}(t)}$ is the unit vector of $R_{0}(t)$ and V is the spatial domain where the reflectivity function $z^{T}$ is defined.

The radial motion compensation can be achieved by removing the phase term $e^{-j\frac{4\pi f}{c}R_{0}(t)}$ and the received signal after motion compensation can be expressed as follows:

(2) $$S_{R}(f,t)=rect(\frac{t}{T_{obs}})rect(\frac{f-f_{0}}{B})\int_{V}\xi (z)e^{-j\frac{4\pi f}{c}(z^{T}•i{}_{R_{0}(t)})}dz$$

The distance $R_{0}(t)$ can be approximated by a polynomial. This can be written as follows:

(3) $$R_{0}(t)\approx \alpha +\beta t+\frac{1}{2}\gamma t^{2}$$

where $\alpha =R_{0}(0)$, $\beta ={\overset{•}{R}}_{0}(0)$ and $\gamma ={\overset{••}{R}}_{0}(0)/2$, $\alpha =R_{0}(0)$ cannot cause the defocusing in the image, the β and γ represent the target radial velocity and acceleration which also are related to the Doppler frequency parameter in (4):

(4) $$\left\{ \begin{array}{l} f_{dc}=\frac{2f}{c}\beta \\ f_{dr}=\frac{4f}{c}\gamma \end{array}\right.$$

The quadratic term $\frac{1}{2}\gamma t^{2}$ is the main cause of defocus in SAR images [3,32].

### 3.2. Phase Compensation Based on Fast Minimum Entropy Method

The features of ship, image entropy and image contrast (IC) are regarded as the evaluation criteria of image focusing. If the image is well-focused, the entropy and IC attain their minimum and maximum values, respectively [25]. Because there are no ground truths of ship features compared with refocused ships, we assume that the geometrical features of the ship are compact for a well-focused ship. The well-focused ships have smaller geometrical features than defocused ships. The geometrical features contain the length, width and the areas of ship.

An ISAR image I(k,n) is a two-dimensional complex image, where k is the range sample number, n is the cross-range number. The entropy of the two-dimensional image is written as follows [33]:

(5) $$E(I)=\sum_{k=0}^{K-1}\sum_{n=0}^{N-1}\frac{{\left|I(k,n)\right|}^{2}}{S}\ln\frac{S}{{\left|I(k,n)\right|}^{2}}$$

where S is the total energy of the image:

(6) $$S=\sum_{k=0}^{K-1}\sum_{n=0}^{N-1}{\left|I(k,n)\right|}^{2}$$

The entropy is relatively small when the image is well-focused, and image refocusing is assessed via Equation (5). The IC denotes the normalized effective power of the image intensity and gives a measure of the image focus. If the image is well-focused, the IC value of the image is large. IC definition is considered as the ratio of the standard deviation to the mean of the amplitude. The IC is written as follows [34]:

(7) $$IC(I)=\frac{\sqrt{E\{{\left[I(k,n)-E\{I(k,n)\}\right]}^{2}\}}}{E\{I(k,n)\}}$$

where E represents the spatial mean operator.

The motion compensation mainly compensates for the translation motion between the target and the radar. It contains two steps, one is range alignment, and the other is phase compensation [8,11]. The raw data-like data R(m,n) is inverted from SLC SAR images and range alignment is a coarse compensation, the raw data-like data R(m,n) no longer implement range alignment and the phase compensation is the main step of ISAR processing in this paper.

Phase compensation and ISAR imaging can be written as follows [23,32,35]:

(8) $$I(k,n)=IFFT_{2D}\{R(k,n)\cdot \exp(-j\phi (m))\}$$

where I(k,n) is the SAR image, ϕ(m) represents the phase error in Equation (2). m is the number of samples in the range direction, n is the cross-range samples number. The key step to phase compensation is estimation of ϕ(m). The phase error estimation $\hat{\phi }(m)$ is obtained by minimizing the entropy of ISAR image:

(9) $$\hat{\phi }(m)=\arg\min E(I)$$

It means that the Equation (9) should be satisfied with (10):

(10) $$\frac{\partial E}{\partial \phi (m)}=0$$

Since the total energy S is constant, the cost function E can be redefined as:

(11) $$E^{\prime }(I)=-\sum_{k=0}^{K-1}\sum_{n=0}^{N-1}{\left|I(k,n)\right|}^{2}\ln{\left|I(k,n)\right|}^{2}$$

Therefore Equation (10) is equivalent to:

(12) $$\frac{\partial E^{\prime }}{\partial \phi (m)}=0$$

The derivation function of the entropy with respect to ϕ(m) is obtained from (13):

(13) $$\frac{\partial E^{\prime }}{\partial \phi (m)}=-\sum_{k=0}^{K-1}\sum_{n=0}^{N-1}\left[1+\ln{\left|I(k,n)\right|}^{2}\right]\frac{\partial {\left|I(k,n)\right|}^{2}}{\partial \phi (m)}$$

Since ${\left|I(k,n)\right|}^{2}=I(k,n)I^{*}(k,n)$, there are:

(14) ∂|I(k,n)|2∂ϕ(m)=2Re(I*(k,n)∂I(k,n)∂ϕ(m))

Then substituting Equation (14) into Equation (13), we have:

(15) ∂E′∂ϕ(m)=−Re∑k=0K−1∑n=0N−1[1+ln|I(k,n)|2]I*(k,n)∂|I(k,n)|∂ϕ(m)

The derivative of I(k,n) with respect to ∂ϕ(m) is acquired as follows:

(16) $$\frac{\partial I(k,n)}{\partial \phi (m)}=-jR(k,n)\exp(-j\phi (m))\exp(-j\frac{2\pi }{M}km)$$

Substituting Equation (16) into (15), one obtains:

(17) ∂E′∂ϕ(m)=−2MIm{exp[−jϕ(m)]w*(m)}

where:

(18) $$w(m)=\sum_{k=0}^{K-1}R^{*}(k,n)R_{l}$$

(19) $$R_{l}=\frac{1}{M}\sum_{m=0}^{M-1}\left[1+\ln\left|I{\left(k,n\right)}^{2}\right|\right]I(k,n)\exp(j\frac{2\pi }{M}km)$$

Finally, Equation (17) is equal to zero, and $\hat{\phi }(m)$ is obtained:

(20) $$\exp(-j\hat{\phi }(m))=\frac{w(m)}{\left|w(m)\right|}$$

### 3.3. The Procedures of Phase Compensation Method

**Figure 4:**
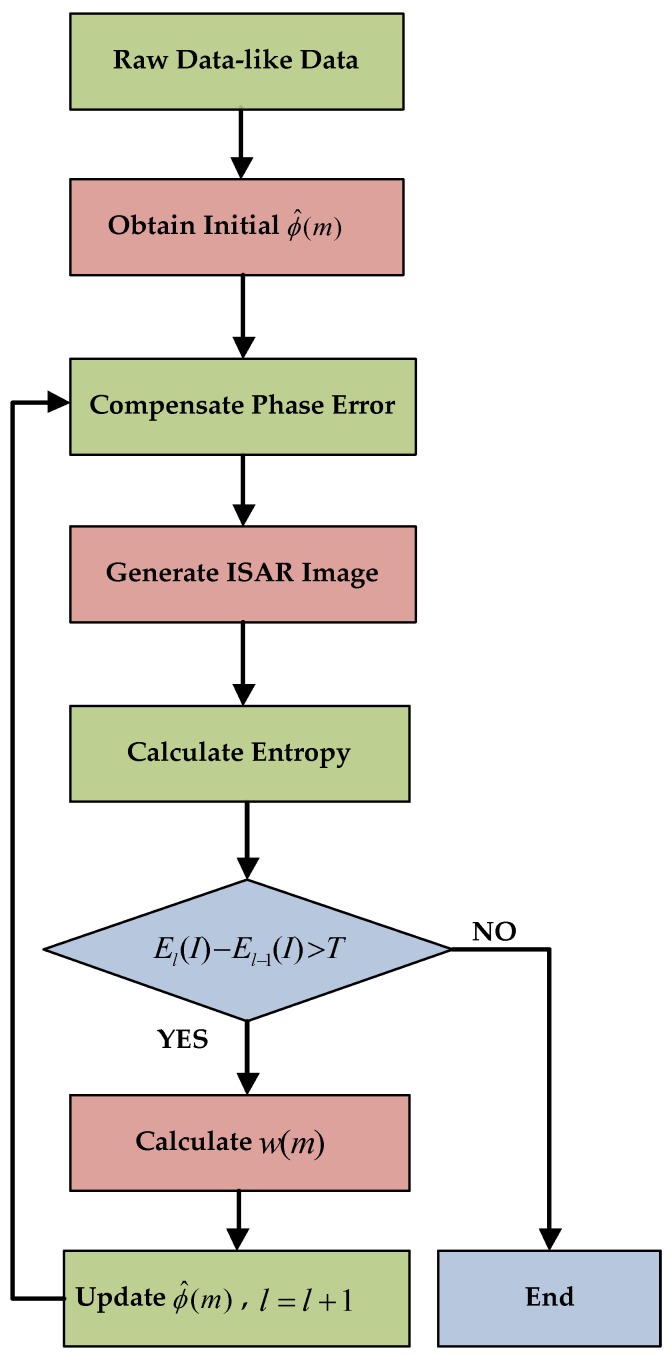
The basic flowchart of phase compensation method based on fast minimum-entropy.

The flowchart of the proposed phase compensation method is shown in Figure 4. The steps of phase compensation method based on fast minimum-entropy are described as follows:

*Step 1*: Input the raw data-like data and utilize DCT method [17] to obtain the initial phase error $\hat{\phi }(m)$, l is the number of iterations.

*Step 2*: Compensate phase error by substituting ϕ(m) with $\hat{\phi }(m)$. Two-dimensional inverse fast Fourier transform is performed to generate the ISAR image.

*Step 3*: Calculate entropy of the ISAR image and set tolerance T which is used to stop the iterations. If $E_{l}(I)-E_{l-1}(I)$ is greater than the T, the iteration continues; otherwise, the process terminates.

*Step 4*: Obtain $R_{l}$ by Fourier transform ln(|I(k,n)|)·$I^{*}(k,n)$ and calculate the w(m).

*Step 5*: Update phase error estimation $\hat{\phi }(m)$ and l=l+1, go back to Step 2.

## 4. Experimental Results and Discussions

Experimental results based on real SAR images are presented in this section to quantitatively evaluate the validity of the proposed method. The experimental data include spaceborne and airborne SAR data. The parameters of the SAR images are presented in Table 1.

The SAR image perform ships detection with software [36] and sub-images containing defocused ship are selected in advance. The raw data-like data is derived from sub-images with the IRD algorithm. The number of iterations l is set at 300. The results of the proposed method are compared with the results of the DCT [17] and PGA [14] methods.

### 4.1. Spaceborne SAR Data

**Figure 5:**
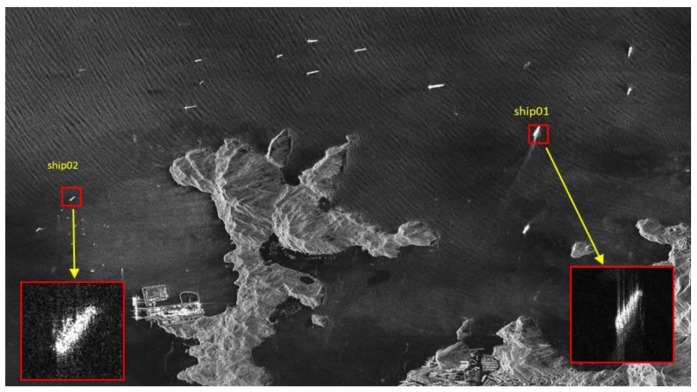
Two defocused ships detected in a TerraSAR-X image (sub-images of ship01 and ship02). The red rectangles are the zoomed sub-images.

The spaceborne SAR data contains data from two kinds of satellite system. One is TerraSAR-X system images, the other is Gaofeng-3 system images. The defocused ship targets of TerraSAR-X images are depicted in Figure 5. The two ships are numbered as ship01 and ship02. The ships are still blurred and defocused due to their motion, and need motion compensation.

**Figure 6:**
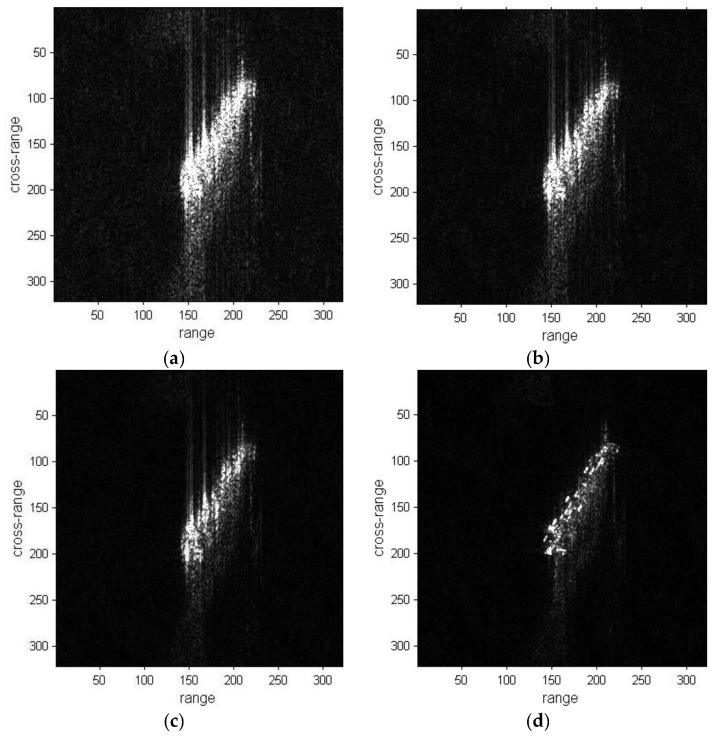
Refocused images of the ship01 sub-image. (**a**) Sub-image of ship01; (**b**) Refocused image with the DCT method; (**c**) Refocused image with the PGA method; (**d**) Refocused image with the proposed method.

**Figure 7:**
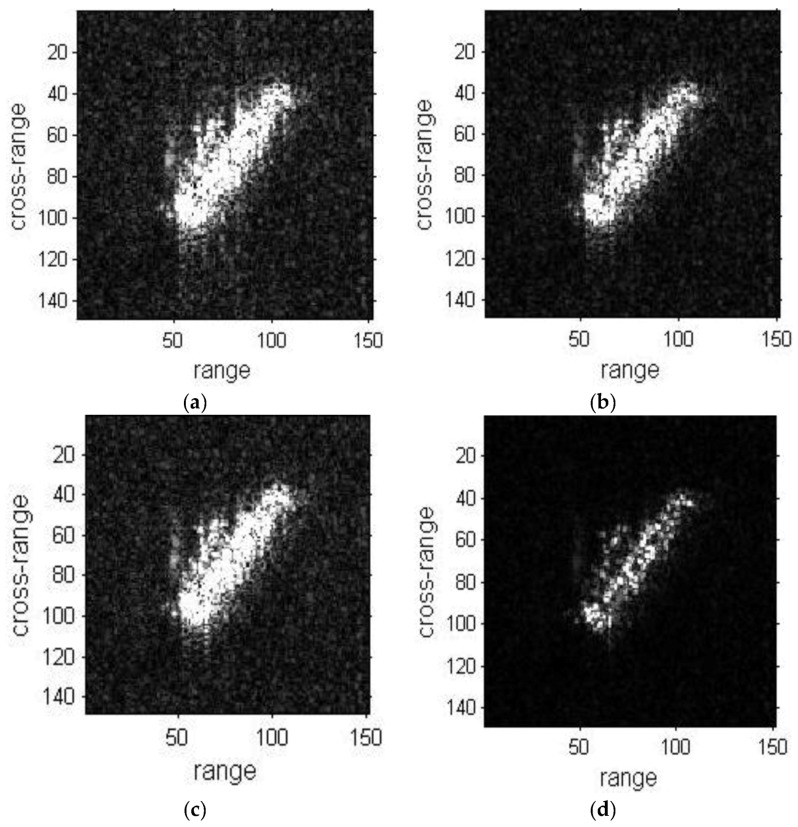
Refocused images of ship02 sub-image. (**a**) Sub-image of ship02; (**b**) Refocused image with the DCT method; (**c**) Refocused image with the PGA method; (**d**) Refocused image with the proposed method.

The refocused results of ship01 with the three methods are shown in Figure 6. The original sub-image, DCT and PGA results are respectively depicted in Figure 6a–c. The result with the proposed method is shown in Figure 6d. The image quality of the proposed method result is better than the original image, DCT and PGA results. The cross-range of the image is well-focused and blurred regions are reduced in Figure 6d. The refocused results of ship02 are shown in Figure 7. The image quality of the proposed method result is also superior to the other results.

**Figure 8:**
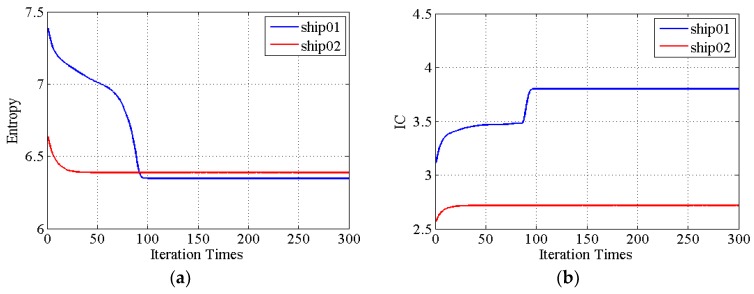
The entropies and IC values convergence of two sub-images versus the number of iterations. (**a**) Entropies change of the two sub-images during iteration; (**b**) IC values change of the two sub-images during iteration.

Image entropy and IC value are the two criteria to assess the image quality. The convergences of the two criteria versus the number of iterations are depicted in Figure 8. As the number of iterations increases, there has been decrease in entropy and increase in IC. Both criteria of ship01 have been greatly improved in Figure 8, and the focusing of the ship01 image is enhanced, while the criteria change of ship02 are not as obvious as for ship01 and the enhancement in refocusing of ship02 is not as good as for ship01.

The geometrical features of the ships are extracted for further assessment. The features include the length, width and area. Since there are no ground truths for the ship features, it is impossible to compare extracted geometrical features with ground truths. The geometrical features of ships in focused images should be smaller than the defocused images.

**Figure 9:**
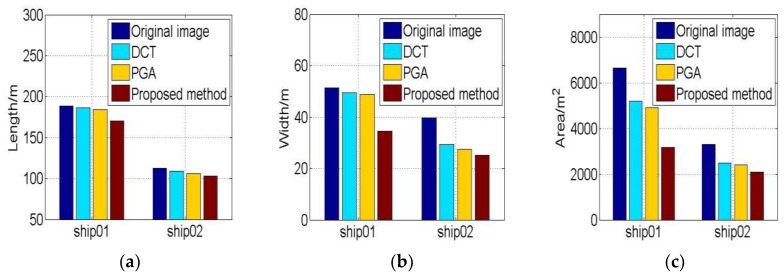
The geometrical features of refocusing ship01 and ship02. (**a**) Lengths of ships; (**b**) Widths of ships. (**c**) Areas of ships.

The extracted features of ship01 and ship02 are illustrated in Figure 9. It is evident that the ship lengths extracted from the proposed method results are smaller than other results in Figure 9a. The widths and areas of proposed method results are also the least of the four results in Figure 9b,c. These results indicate that the refocusing result of the proposed method is better than that of the other methods.

**Figure 10:**
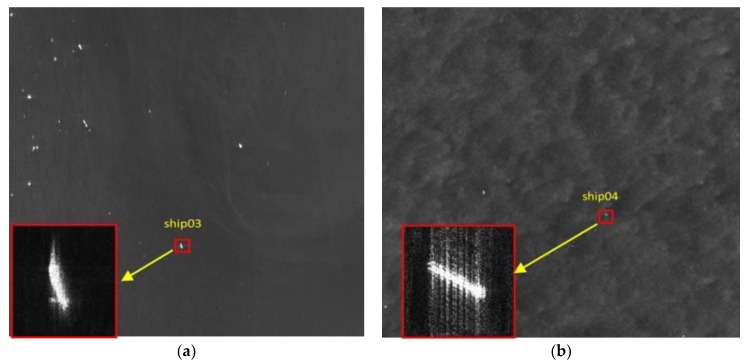
Two defocused ships are detected in Gaofeng-3 images. (**a**) Sub-image of ship03 located in the Gaofeng-3 image; (**b**) Sub-image of ship 04 located in the Gaofeng-3 image. The red rectangles are the zoomed sub-images.

The defocused ships detected in two Gaofeng-3 SAR images are illustrated in Figure 10. The two ships are numbered as ship03 and ship04, respectively. It is obvious that the two ships are blurred and defocused in the SAR images.

**Figure 11:**
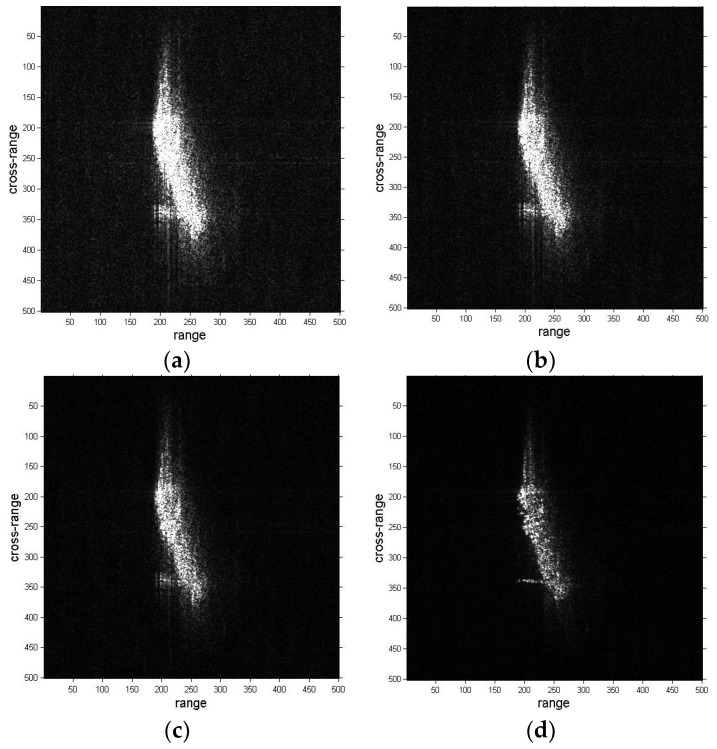
Refocused images of ship03 sub-image. (**a**) Sub-image of ship03; (**b**) Refocused image with DCT method; (**c**) Refocused image with PGA method; (**d**) Refocused image with proposed method.

The refocusing results of ship03 are presented in Figure 11. The result of the proposed method is better than the DCT and PGA method results in Figure 11d. The left side regions of the ship are obviously well-focused and become clear in Figure 11d. 

**Figure 12:**
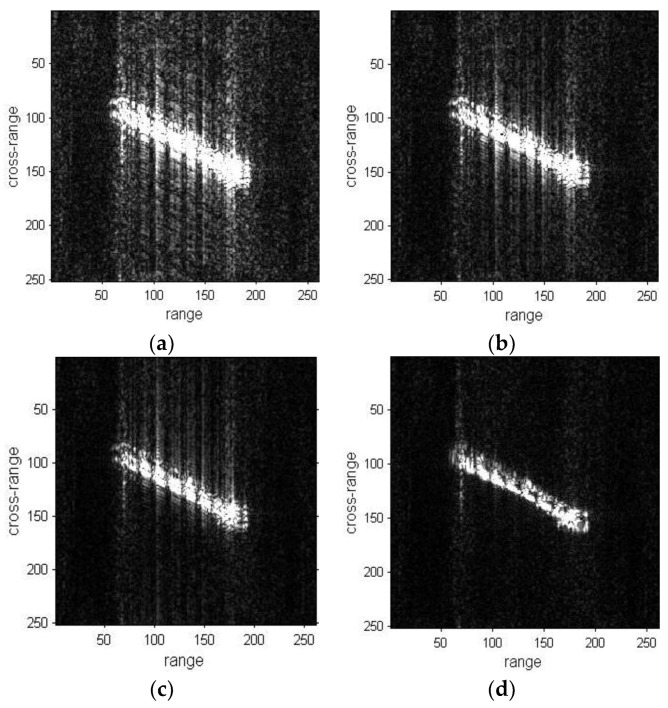
Refocused images of ship04 sub-image. (**a**) Sub-image of ship04; (**b**) Refocused image with DCT method; (**c**) Refocused image with PGA method; (**d**) Refocused image with proposed method.

The results of ship04 are shown in Figure 12, and the results of DCT, PGA and the proposed method are presented in Figure 12b–d, respectively. The focusing of DCT and PGA results produce a certain improvement compared with the original images, but are not well-focused. The blurring in the cross-range direction is greatly removed in Figure 12d. As stated previously, the image quality of the proposed method results are superior to the original images, DCT and PGA results from a visual point of view.

**Figure 13:**
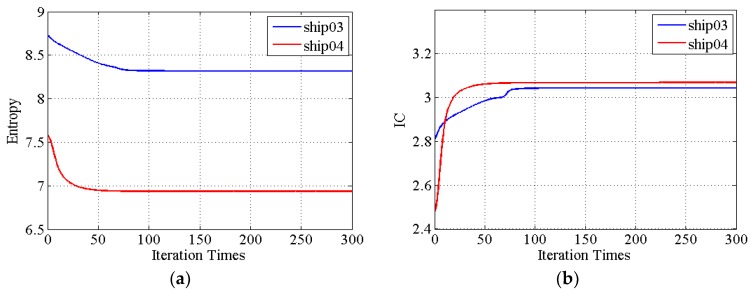
The entropies and IC values convergence of two sub-images versus the number of iterations. (**a**) Entropy change of the two sub-images during iteration. (**b**) IC values change of the two sub-images during iteration.

**Figure 14:**
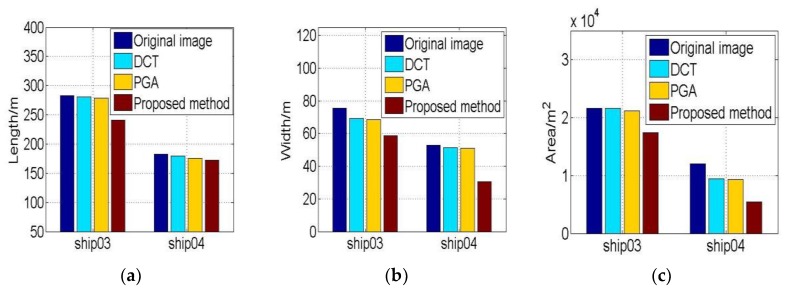
The geometrical features of refocusing ship03 and ship04. (**a**) Lengths of ships; (**b**) Widths of ships. (**c**) Areas of ships.

The entropies and IC values during iteration are represented in Figure 13. The entropies are getting smaller and IC values larger with iteration, showing that the quality of the image is improved. The entropies and IC values of ship04 are changing greatly, demonstrating that the image quality of ship04 sub-image processing with the proposed method is good. The geometrical features of ship03 and ship04 are shown in Figure 14. The refocusing feature results with the proposed method are also smaller than the other method results.

### 4.2. Airborne SAR Data

**Figure 15:**
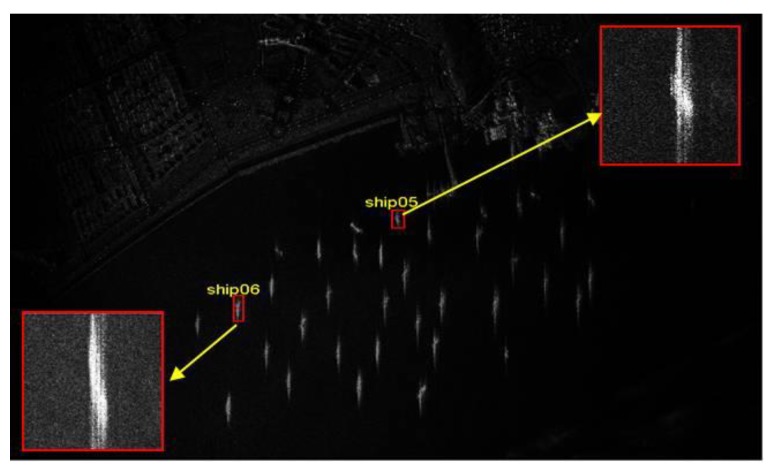
Two defocused ships are detected in airborne SAR images. Sub-images of ship05 and ship06 are located in airborne SAR image. The red rectangles are the zoomed sub-images.

In addition to spaceborne SAR data, an airborne SAR dataset is exploited to further validate the procedure. The defocused ships are marked in the airborne SAR image in Figure 15. The two ships are numbered as ship05 and ship06. The resolution of the airborne SAR image is higher than that of the spaceborne SAR images.

The defocusing of moving ship in airborne SAR image is more evident. The Doppler rate shift induced by target motion in the airborne SAR system is greater than in the spaceborne SAR system. The Doppler rate has great influence on the image quality and a little error will result in image defocusing and decreased resolution. The degree of defocusing is directly related to the Doppler rate shift [5].

**Figure 16:**
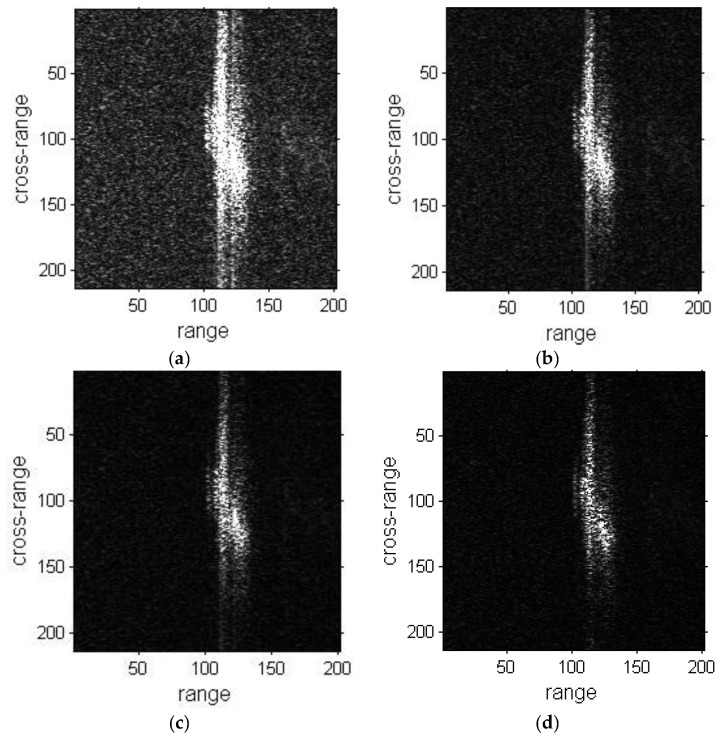
Refocused images of ship05 sub-image. (**a**) Sub-image of ship05; (**b**) Refocused image with the DCT method; (**c**) Refocused image with the PGA method; (**d**) Refocused image with the proposed method.

**Figure 17:**
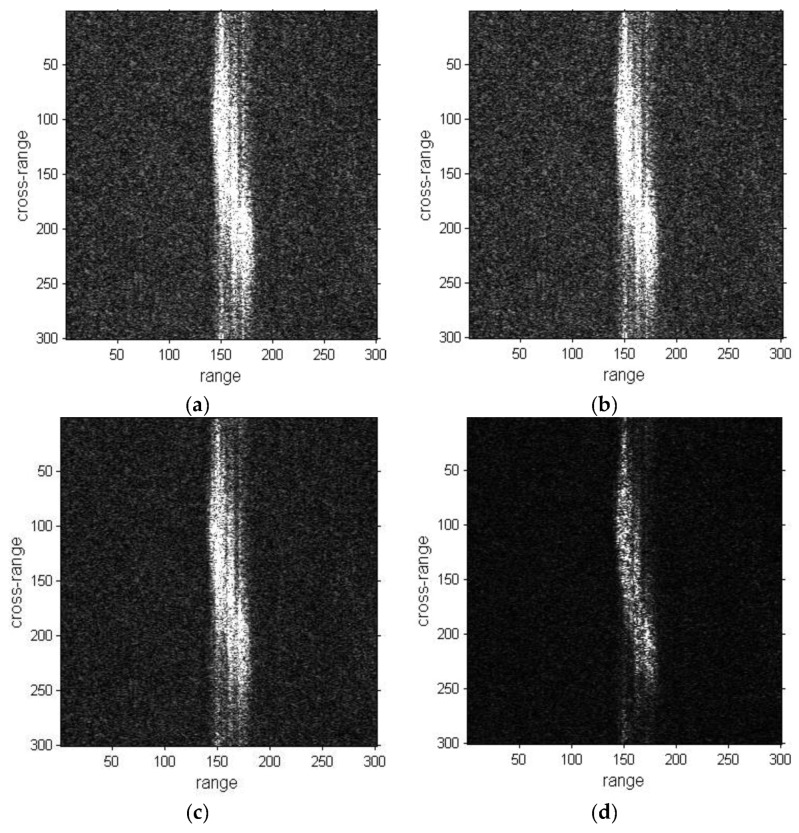
Refocused images of ship06 sub-image. (**a**) Sub-image of ship06; (**b**) Refocused image with the DCT method; (**c**) Refocused image with the PGA method; (**d**) Refocused image with the proposed method.

The experimental results of ship05 are shown in Figure 16. As can be noted by observing Figure 16d that the refocused result with the proposed method has some improvement in focusing compared with the original images, DCT and PGA results. The results of ship06 are illustrated in Figure 17. The images with ISAR processing all have certain improvement in focusing compared to the original image. The result of the proposed method is still better than the DCT and PGA method results.

The ISAR processing is not able to produce a well-focused target from airborne SAR images and the refocusing are not as good as for spaceborne SAR sub-images. The reason is that the IRD inversion is applied in SAR images for which the resolution is of the order of meters, while the resolution of airborne SAR images is higher than one meter. The inversion of high-resolution SAR images (higher than 1 m) would be need a more accurate inversion algorithm.

**Figure 18:**
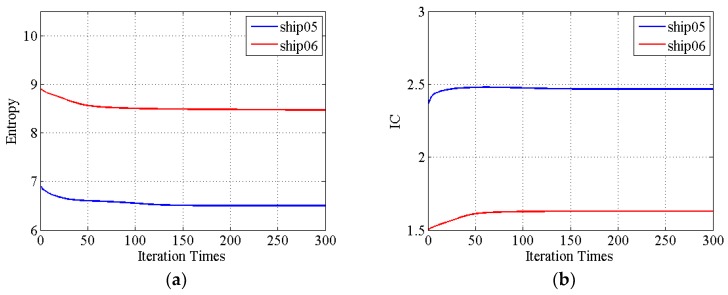
The entropies and IC values convergence of two sub-images versus the number of iterations. (**a**) Entropies change of the two sub-images during iteration; (**b**) IC values change of the two sub-images during iteration.

**Figure 19:**
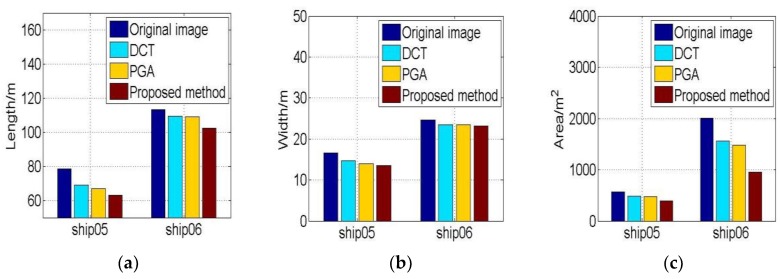
The geometrical features of refocusing ship05 and ship06. (**a**) Lengths of ships; (**b**) Widths of ships. (**c**) Areas of ships.

The entropies and IC values change are illustrated in Figure 18. The entropy and IC value of the proposed method results have some changes with iteration. This means the image quality shows some improvement. The features of ship05 and ship06 are shown in Figure 19. The features of the proposed method result are the least yet.

The entropies and IC values of the original and refocused images are calculated for quantitative evaluation of the focusing. The performance of the proposed method is compared with the DCT, and PGA methods and the original images. The entropies of sub-images with different method processing are presented in Table 2. All entropies of the proposed method results are lower than others in the table. This means that the focusing of the proposed method result could outperform the other method results. The IC values of the sub-images are presented in Table 3. The IC values of the proposed method results are quite larger than the other results, particularly for spaceborne sub-images.

## 5. Conclusions

By exploiting the ISAR technique based on fast minimum entropy phase compensation, a refocusing method for moving ships in SAR images is proposed in this paper. The basic procedures of refocusing in SAR images are built. The processing data are SAR images rather than raw echo data. The algorithms inverting images to the raw data domain are described. This makes the processing flow simple and reduces the computational burden. The ISAR processing based on the fast minimum entropy phase compensation method iteratively obtains the phase error estimation by constructing a cost function of entropy. The experimental results based on spaceborne and airborne SAR data verify the effectiveness of the proposed method.

Though the experimental results are good, the airborne SAR sub-images (resolution higher than 1 m) are not well focused via ISAR processing. The high-resolution SAR image inversion needs to be investigated in future work.

## Figures and Tables

**Table 1:** The parameters of four SAR images.

Image	Image01	Image02	Image03	Image04
Product	TerraSAR-X	Gaofeng-3	Gaofeng-3	Airborne
Mode	Strip	UFS	UFS	Strip
Resolution (M)	3	3	3	0.5
PRF (Hz)	3472.134984	2014.078491	1977.984863	500.0000
Band (MHz)	120.00	80.00	80.00	150.00
Polarization	VV	HH	HH	HH
Wave Length (m)	0.031040	0.055517	0.055517	0.056564
Slant-Range (km)	629.17	7127.22	7137.52	4.62
Velocity (m/s)	7088.636524	7563.162316	7568.372931	55.599743

**Table 2:** The entropies of six sub-images.

Sub-Image	ship01	ship02	ship03	ship04	ship05	ship06
**Original Image**	7.39	6.59	8.72	7.61	6.92	8.92
**DCT**	7.26	6.54	8.69	7.58	6.89	8.89
**PGA**	7.05	6.51	8.64	7.50	6.81	8.76
**Proposed Method**	6.35	6.30	8.32	6.94	6.50	8.45

**Table 3:** The IC values of six sub-images.

Sub-Image	ship01	ship02	ship03	ship04	ship05	ship06
**Original Image**	2.97	2.42	2.81	2.44	2.33	1.49
**DCT**	3.11	2.50	2.85	2.48	2.37	1.50
**PGA**	3.25	2.54	2.88	2.56	2.38	1.53
**Proposed Method**	3.80	2.67	3.04	3.07	2.47	1.63
